# Home-Made Lateral Flow Test Strip Versus POC-CCA Assay for Detection of Active Schistosomiasis in Egypt

**DOI:** 10.1007/s11686-024-00917-9

**Published:** 2024-10-02

**Authors:** Manal Kamel, Faten Salah, Zeinab Demerdash, Sara Maher, Hanan El-Baz, Nahla Zakaria, Rania Abu-Shady, Amany Saad, Salwa Hassan, Doaa Abdel Aziz

**Affiliations:** 1 Immunology Department, Theodor Bilharz Research Institute, Kornish El Nil Street, Giza, Egypt; 2 Clinical Pathology, Faculty of Medicine, Ain Shams University, Cairo, Egypt

**Keywords:** Schistosomiasis, Monoclonal antibodies, Nanoparticles, Lateral flow test

## Abstract

**Background:**

For years, the Kato-Katz (KK) technique has been considered the gold standard for diagnosing schistosomiasis. The aim of this study was to compare the effectiveness of our previously developed gold nanoparticle-based lateral flow test strip (AuNPs-LFTS) for diagnosing active *Schistosoma mansoni* with that of the commercially available point-of-care Circulating Cathodic Antigen detection (POC-CCA) kit.

**Methods:**

In this study, we collected sixty positive and twenty negative urine samples from patients in endemic hot spots in the Nile Delta, as well as from patients visiting the internal medicine clinic at Theodor Bilharz Research Institute (TBRI). We produced monoclonal antibodies (MAbs) against *S. mansoni* soluble egg antigen (SEA) from cloned hybridoma cells (4D/1D). These MAbs were conjugated with gold and mesoporous silica nanoparticles, and used to develop the LFTS.

**Results:**

The LFTS demonstrated a limit of detection (LoD) of 3 ng/ml. The sensitivity and specificity of the developed LFTS were found to be 96.7% and 95%, respectively, compared to 85% and 90% for the POC-CCA detection kit. The cases were divided into groups based on egg count in the stool, categorized as light, moderate, and heavy infections. The sensitivity of the LFTS in the group with light infection was higher than that of the POC-CCA. When using the KK technique (eggs per gram of stool sample [EPG]) as the reference test, the kappa value for the nano-based strips was 0.902, compared to 0.672 for the CCA strips, indicating an almost perfect agreement between KK and our developed LFTS.

**Conclusion:**

These results confirm the reliability and effectiveness of the LFTS compared to commercially available kits for rapid, sensitive, and early diagnosis of schistosomiasis. However, it is recommended to conduct further assessments of the developed strip on a larger scale with a broader range of cases before considering its introduction to local or international markets.

**Graphical Abstract:**

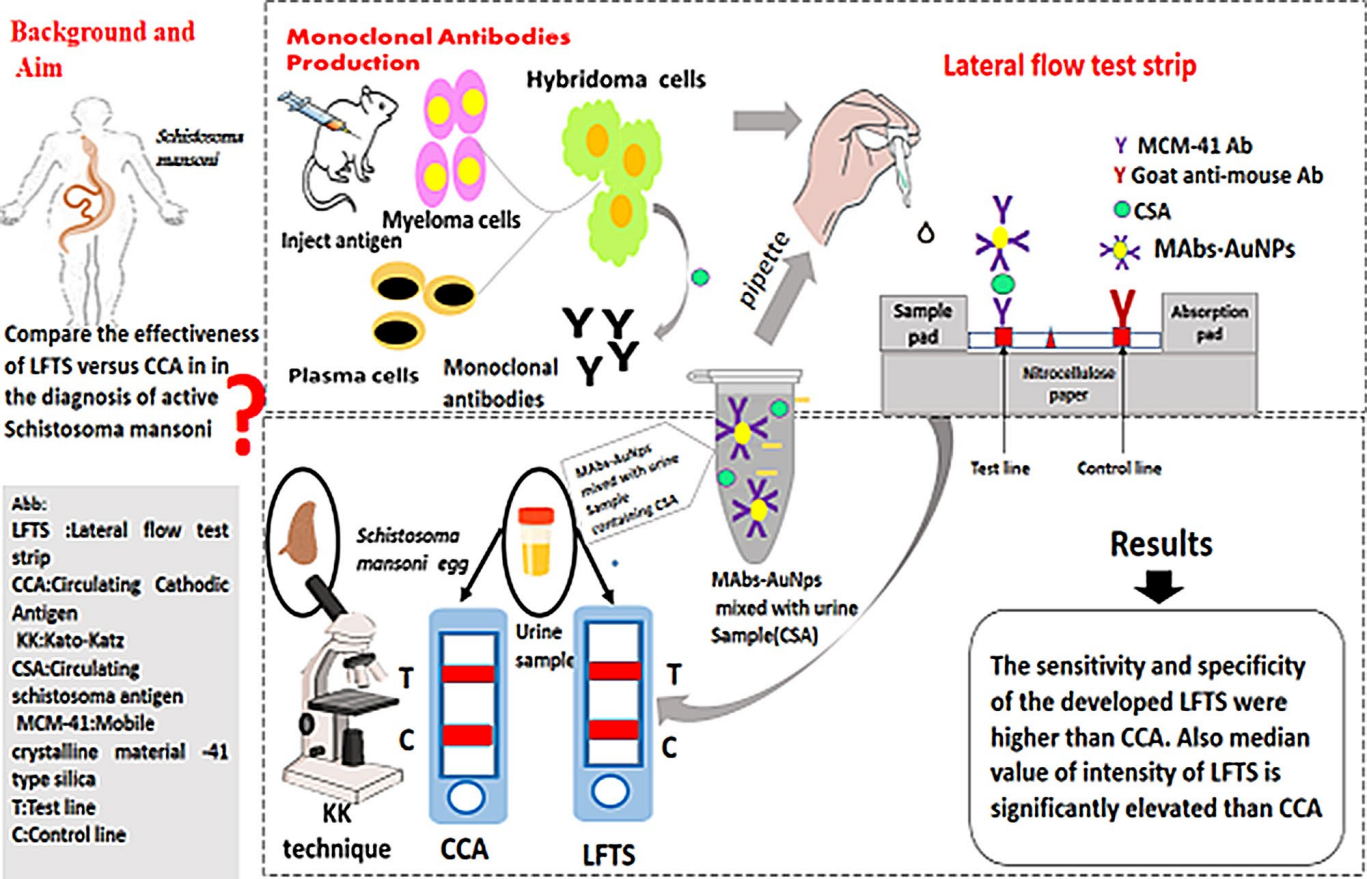

## Introduction

Schistosomiasis is one of the major neglected health problems in Africa, affecting nearly 220.8 million people in 78 countries worldwide, necessitating treatment. Early diagnosis is crucial for effective control programs and preventive treatment to reduce and prevent morbidity [1]. The Kato-Katz (KK) method is considered the gold standard for diagnosing schistosome eggs in stool samples. However, it has several limitations [2, 3]. Despite years of research, there has been a continuous pursuit to find a rapid and sensitive one-step diagnostic test for schistosomiasis as an alternative to conventional microscopic detection methods. Since 1995, researchers at Theodor Bilharz Research Institute (TBRI) in Cairo, Egypt, have developed a panel of monoclonal antibodies using hybridoma technology against various *Schistosoma* antigens [4, 5]. In several studies, monoclonal antibodies were employed in a sandwich enzyme-linked immunosorbent assay (ELISA) to detect circulating antigens in urine and serum samples as diagnostic probes for early active schistosomiasis infections. While this method improved the sensitivity and specificity of diagnosis and overcame the drawbacks of previous conventional methods, it was not practical for routine screening due to its multiple steps, long reaction time, and limited suitability to well-equipped laboratories [6, 7].

Recently, there has been growing interest in the use of immune-strip assays as an alternative method, which offers a one-step, user-friendly approach with a visual endpoint [8]. The detection of circulating cathodic antigen (CCA) using urine strips has been widely applied for routine *S. mansoni* infection detection [9]. In 2004, van Dam et al. reported that CCA detects a specific parasite gut carbohydrate antigen regurgitated by adult worms, which circulates in the bloodstream, is eliminated by the kidneys, and can be detected in the urine of infected individuals [10]. However, ELISA still demonstrated higher sensitivity than CCA, resulting in multiple false positive results. Many studies have focused on improving CCA, and researchers have explored the use of colloidal gold nanoparticles (AuNPs) to enhance the sensitivity of conventional immune-strip assays, enabling rapid and cost-effective detection of infectious agents compared to current available technologies [11–14]. In 2016, Kamel et al. found that loading monoclonal antibodies onto gold nanoparticles increased the specificity and sensitivity of monoclonal antibody-based sandwich ELISA to 100% and 98.7%, respectively [15].

Nanomaterials can enhance the performance of diagnostic assays by providing additional binding sites for detection antibodies. This leads to an increase in signal intensity. Gold nanoparticles, in particular, are effective colored reporters due to their high color intensity, which can improve the sensitivity of lateral flow tests (LFTs) [16] Additionally, gold nanoparticles have other favorable characteristics, such as cost-effectiveness, ease of production, stability in dried form, and the ability to be easily conjugated with biomolecules. These properties make gold nanoparticles an ideal choice as signal reporters in industrial applications [12, 17]. Additionally, mesoporous silica nanoparticles (MSN) of the mobile crystalline material (MCM)-41 type offer efficient immobilization of proteins on nitrocellulose membranes, ensuring good efficiency, immune reactivity, and stability of the immobilized protein [18].

In 2019, Kamel et al. developed a convenient and sensitive gold nanoparticle-based lateral flow test strip (LFTS) assay for rapid detection of soluble egg antigen (SEA) from *S. mansoni* in serum and urine samples of schistosomiasis-infected patients, utilizing colloidal AuNPs and MSN. After large-scale production, purification, and characterization of the produced monoclonal antibodies (4D/1D), the LFTS was constructed using gold-conjugated detector monoclonal antibodies and MCM-41-conjugated capture monoclonal antibodies for immobilization on the nitrocellulose membrane. Through optimization and standardization of working conditions, we tested the developed strip using serum and urine samples from infected patients. Our previous results were very promising, with a specificity of 97.5% and sensitivity of 98.3% in urine [5].

## Study Design and Sample Collection

This study aims to compare the effectiveness of the developed LFTS with the commercially available POC-CCA kit for the rapid detection of *S. mansoni* SEA in urine samples of infected patients as a validation step for large-scale application in routine diagnosis. Samples were collected from endemic hotspots in the Nile Delta, specifically Elkhamseeny and Sandala villages in Kafr Elsheikh Governorate. Three hundred (300) stool samples were screened using the KK technique for *S. mansoni* infection. Only 60 cases tested positive and were included in this study, along with 20 negative cases used as a control group of matched sex and aged from 12 to 30 years.

The positive cases were subdivided according to the number of eggs per gram (EPG) into the light infection group (12 cases) (< 50 EPG), moderate infection group (11 cases) (51–100 EPG), and heavy infection group (37 cases) (≥ 100 EPG). Urine samples from all included subjects were collected, centrifuged for 5 min at 2000 rpm, and then the supernatant was collected and stored at -20 °C until used.

## Methods

### Production of S.mansoni SEA-mAbs

Hybridoma cells secreting mAbs (4D/1D), which were raised against *S. mansoni* SEA, were developed and cryopreserved at the Immunology Department of TBRI in Cairo, Egypt. The mAbs (4D/1D) were characterized as IgG1 kappa-type light chain antibodies that recognize repetitive epitopes on SEA, allowing their use as both antigen-capturing and detecting antibodies in sandwich assays. The reactivity of the hybridoma cells against SEA was checked by indirect ELISA after revival and propagation in a culture medium. Large-scale production of mAbs was maintained by injecting hybridoma cells (2 × 106/ml) intraperitoneally into BALB/c mice to develop ascitic fluid. The produced mAbs were then purified by ammonium sulfate precipitation according to Nowotny [19], followed by treatment with caprylic acid according to McKinney and Parkinson [20].

### Preparation of Nanoparticles Conjugated mAbs

Purified mAbs were passively conjugated with AuNPs according to Tanaka et al. In brief, 30 µg (12 µl) of mAbs solution (2.5 mg/ml) was diluted with KH2PO4 solution (5 mM) in ultra-pure water (200 µl) at pH 7.5. The diluted solution was added to AuNPs (1.8 µl, 20 nm) and immediately mixed, then left to rest for 20 min at room temperature (RT). A blocking step was performed using 200 µl of 10% bovine serum albumin (BSA w/v), followed by centrifugation at 4 °C for 10 min at 8000 g. Pulse sonication for a few seconds was applied, and the conjugated mAb-AuNPs were added to 0.05% and 20 mM Tris-HCl buffer, 2 ml of preserving solution (pH 8.2, 1% [w/v] BSA), and then stored at 4 °C until used [11]. The conjugation step of mAbs with MCM-41 type silica nanoparticles was performed according to Omidfar et al. In brief, 1 mg of MCM-41 was dispersed in 1 ml of phosphate buffer solution (PBS) (pH 7.2, 0.1 M). Then, 181.5 µl of the MCM-41 solution was added to 18.1 µl (45.3 µg) of mAb (4D/1D) (2.5 mg/ml) in a 4:1 ratio. The mixture solution was left at 4 °C overnight. A blocking step for non-specific binding sites was performed by adding 200 µl of 10% BSA (w/v) in PBS (0.1 M) at pH 7.2. The solution was then centrifuged at 4 °C for 4 min, the supernatant was discarded, and the sediment pellet was dispersed in PBS (pH 7.2, 0.1 M). The MCM-41-mAb conjugate was stored at 4 °C before use [21].

### Fabrication of the AuNPs-LFTS

**Fig. 1:**
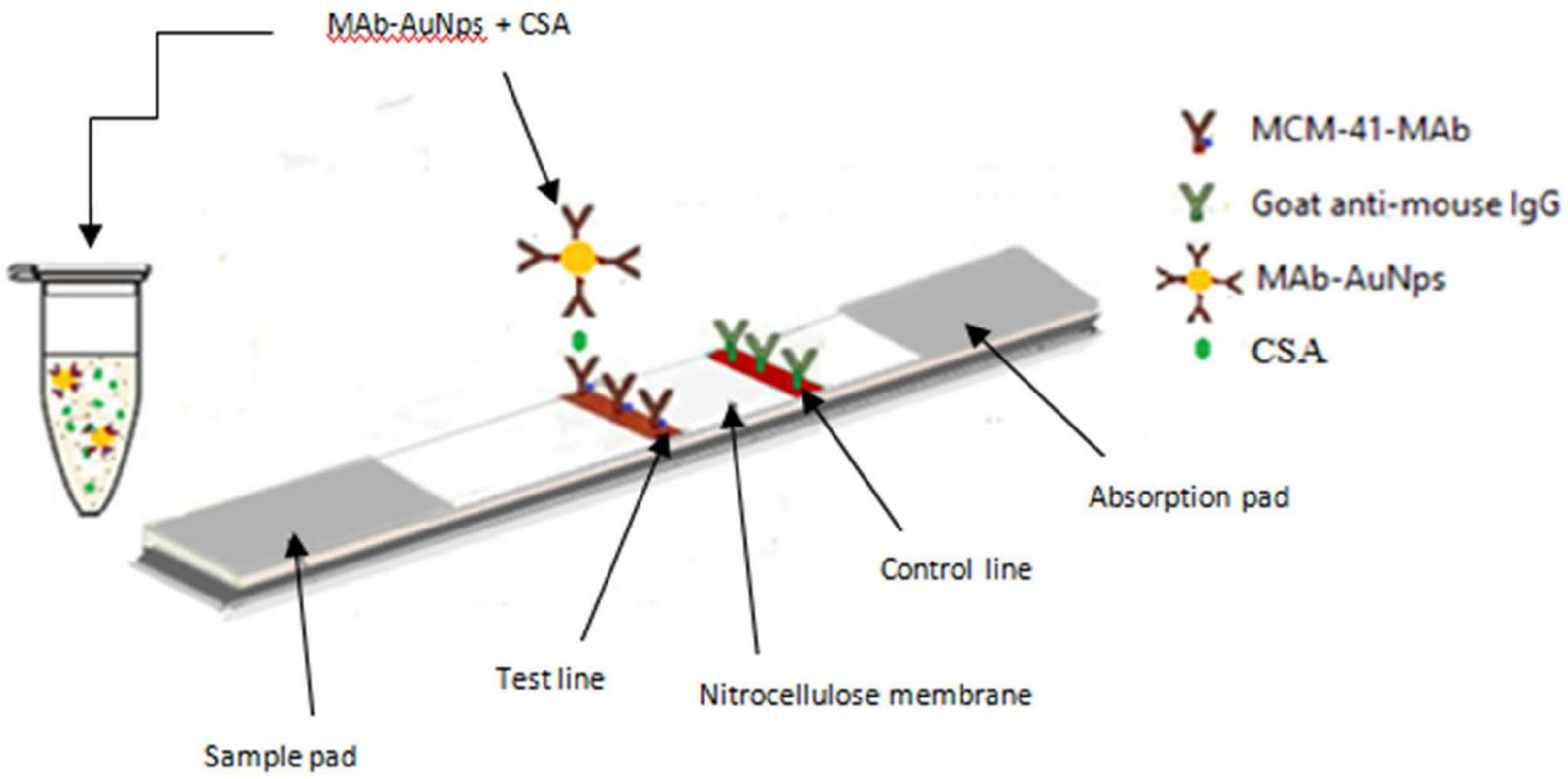
Illustration for the principle of the sandwich LFTS for detection of active *S.mansoni*. In a vial, AuNps-mAb was mixed with urine sample containing circulating *Schistosoma mansoni*

Fabrication of the LFTS was conducted according to our previous work by Kamel et al. (2019) In brief, the test strip is composed of a nitrocellulose membrane, sample pad, and absorbent pad. Several optimization trials were performed before determining the standard conditions of the test [19]. Test line and control line solutions were prepared and standardized. For this patch, the optimal concentration of the capture conjugate (MCM-41-mAbs) on the test line was found to be 5 µl/strip. A control line solution (anti-mouse IgG, 2 mg/ml) was also prepared and dispensed on the nitrocellulose membrane (3 µl/strip). The nitrocellulose membrane was dried at room temperature for 1 h, then immersed in a 50 mM boric acid buffer (skim milk [0.5% [w/v], pH 8.5) for 25 min to block nonspecific adsorption. Finally, the membrane was cut into specific sizes (0.5 cm wide, 6 cm long), and some strips were placed into plastic housings. All strips were stored in a plastic bag at 37 °C until use. The urine sample was mixed with the detector probe (AuNPs-mAbs) in an external tube at a ratio of 10:1 (50 µl of urine sample to 5 µl of AuNPs-mAbs) antigen (CSA), then it was migrated through the nitrocellulose membrane and captured by MCM-41-mAb at the test line forming a distinct red color (Fig. 1).

### Application of Sandwich Based LFTS

Urine samples from the studied groups were subjected to the LFTS assay to detect the presence of circulating *S. mansoni* antigen. All urine samples were also assayed in parallel using 96-well microtiter plates. Briefly, 5 µl of the AuNPs-mAbs conjugate (detector probe) and 50 µl of each urine sample were mixed in separate wells. The test strip’s sample pad was then immersed in the well, allowing the mixture to be absorbed and moved by capillary force, forming a complex with the MCM-41-mAbs employed as the capture probe on the test line. In the presence of the antigen, a red color will appear. The control line will turn red after binding with the anti-IgG antibody to any unbound free conjugates. The test results were obtained after 10 min. The test result is considered invalid if no red color appears on the control line, regardless of the color on the test line.

### Application of POC-CCA

A point-of-care circulating cathodic antigen detection kit (Rapid Medical Diagnostics, Pretoria, South Africa, RSA) was used to test urine samples from the studied groups for the presence of circulating *S. mansoni* antigen. Briefly, 100 µL of urine (equivalent to 2 drops) were transferred to the test cassette by gently squeezing the pipette. The sample was completely absorbed into the specimen pad within the circular well, and then 1 drop of buffer was added. The test results were read after 20 min. The intensity of the red color on the produced test line for both strips was visually assessed using a gel documentation system (Gel Doc XR+, Biorad, USA). The results were then analyzed using Image Lab software version 3.

### Statistical Analysis

Statistical analyses were performed by using the analytical software package IBM-SPSS (version 23). Characteristics of LFTS strip and CCA assays were tested by using receiver operating characteristic curve (ROC). CCA strip and nano-based strip were compared to the EPG test (reference test) based on the following accuracy measure: specificity, sensitivity and Cohen’s kappa statistic (κ) of agreement. According to Kolmogorov-Smirnov test, the data of the intensities of CCA strip and LFTS were not normally distributed. Accordingly, non-parametric tests were used to statistically analyze the data. The association in results between reference test and the immuno-strip assays was detected by Chi-square test. Statistical differences in the intensities between positive and negative groups were compared by using Mann-Whitney U test.

## Results

### Kato-Katz Technique and Infection Intensity

Based on the KK technique, the level of infection intensity was classified as light (12 cases), moderate (11cases), or heavy (37 case), with thresholds set at < 50 EPG, 51–100 EPG, and ≥ 100 EPG, respectively.

### Comparative Accuracy Measures of LFTS and POC-CCA Strip

Upon application of both assays to detect CSA (target antigen) in urine samples of subjected groups, we observed that 58 out of 60 positive KK confirmed cases were found positive by LFTS while 19 out of 20 of the control group were confirmed negative demonstrating a sensitivity and specificity of 96.7% and 95% respectively. For the POC-CCA assay, 51 out of 60 *S.mansoni* infected cases were positive while 18 out of 20 control cases were negative exhibiting sensitivity and specificity of 85% and 90% respectively. Furthermore, the kappa value of LFTS with EPG (as a reference method) was 0.902, reflecting an almost perfect agreement between the two assays. On the other hand, POC-CCA strips showed a substantial agreement with EPG where the kappa value was o.672 (Table 1).

**Fig. 2:**
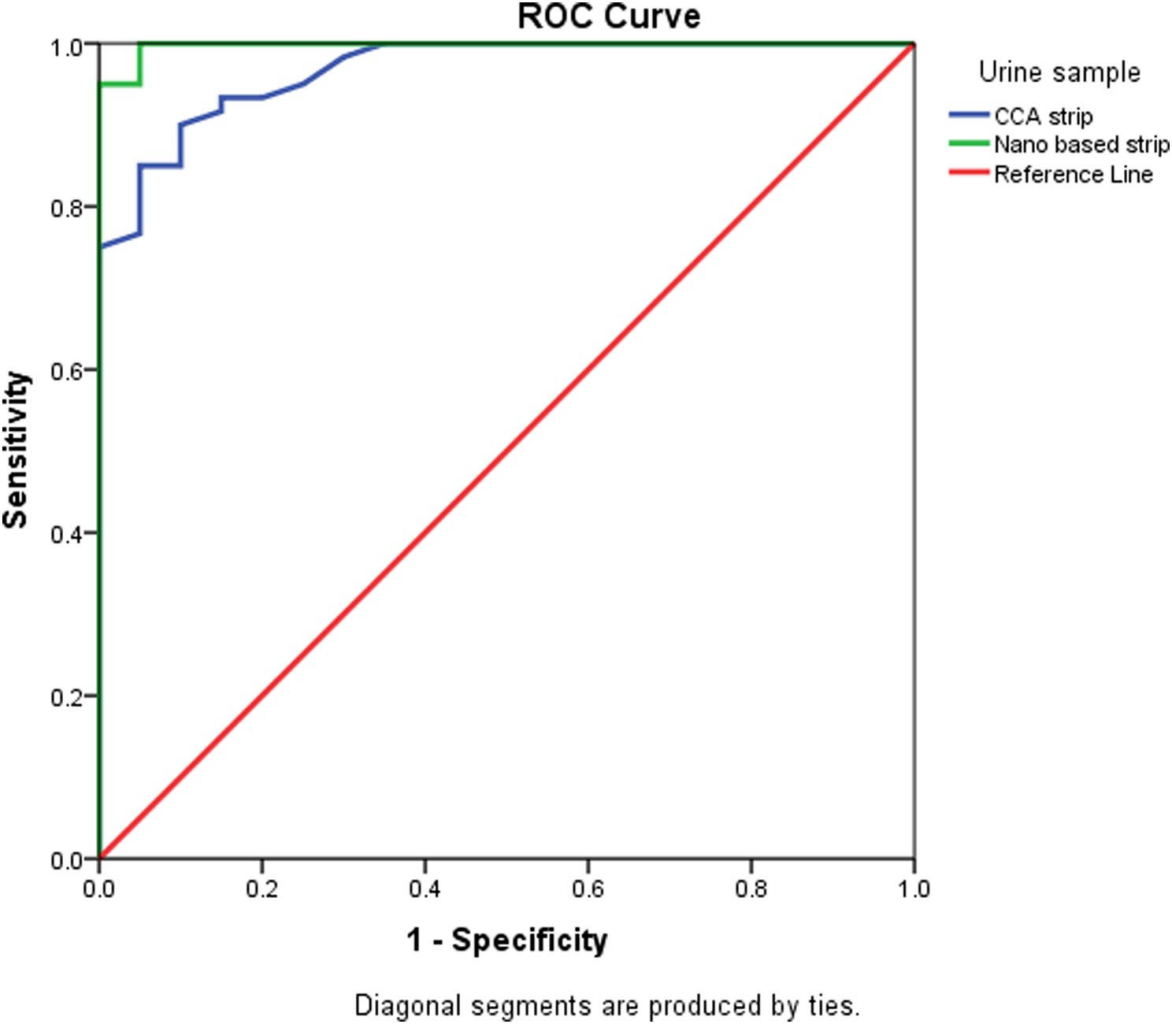
ROC curve of LFTS and CCA strip intensities using EPG as the reference test. The best cutoff values for LFTS and CCA strip are 0.68 and 0.73, respectively

**Fig. 3:**
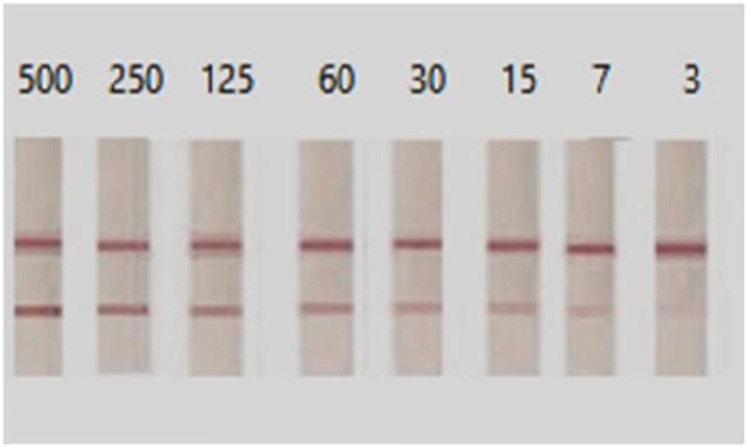
Determination of the visual detection limit of LFTS by using different concentrations of SEA (starting from 500 ng/ml down to 3 ng/ml). The LFTS had a detection limit of 3 ng/ml for SEA

Gel documentation system was used to quantitative measure the color intensity of the test line for both assays, where the intensity is directly proportional to the concentration of the antigen in the tested samples. Intensities were displayed as a volume x10^5^ using Image Lab software. The receiver operating characteristic (ROC) curve was used for the detection of intensities of both LFTS and CCA strips using the same urine samples with EPG as a reference test is displayed in Fig. 2. According to ROC analysis, in CCA strips, the area under the curve (AUC) was found 0.968, with an optimal cut-off value of 0.73. On the other hand, AUC in LFTS strips was 0.998, with a 0.68 optimal cutoff value indicating that both strips have a high ability to discriminate the positive from negative cases. However, the sensitivity and specificity of the LFTS strip were always higher than that of the CCA strip, using EPG as the reference test. Moreover, the color intensity of the test line in the positive cases and their accuracy measures using both strips are displayed in Fig. 3; Tables 2 and 3. Using LFTS, only 2 out of 60 cases were negative and belonged to the light infection group (L). In contrast, 9 out of 60 cases showed negative results when using CCA, encompassing the light (L), moderate (M), and heavy (H) infection subgroups. The sensitivity of LFTS was higher than that of CCA across all infected subgroups, including L, M, and H infections, with a specificity of 100% in the M and H infection groups and 90% in the L infection group. In comparison, the CCA test exhibited specificity of 82%, 82%, and 95% for L, M, and H infections, respectively.

**Table 1:** The accuracy measures of LFTS and CCA strips for CSA detection in urine samples using EPG as a reference test

Strips	Strip/ EPG (-/-)	Strip/ EPG (+/+)	Specificity	Sensitivity	Kappa value	χ2	df	*p*-value
LFTS	19/20	58/60	95%	96.7%	0.902	65.4	1	0.000
CCA	18/20	51/60	90%	85%	0.672	37.7	1	0.000
	AUC	P-value	95% confidence interval	Cutoff value	AUC	P-value	95% confidence interval	Cutoff value
LFTS	0.998 ± 0.003	< 0.000	0.991-1.000	0.68	0.998 ± 0.003	< 0.000	0.991-1.000	0.68
CCA-strip	0.968 ± 0.017	< 0.000	0.933-1.000	0.73	0.968 ± 0.017	< 0.000	0.933-1.000	0.73

**Table 2:** Color intensities of positive *S. mansoni* cases using LFTS and CCA

	CCA Color intensity		LFTS Color intensity	
	Negative	Positive	Negative	Positive
Number of cases	9	51	2	58
Total (60)	0.74 ± 0.02	1.70 ± 0.09^***^	0.74 ± 0.01	2.37 ± 0.10
Subgroups				
L (*n* = 12)	0.75 ± 0.02 (4)	1.03 ± 0.07 (8)^*^	0.74 ± 0.01 (2)	1.64 ± 0.10 (10)
M (*n* = 11)	0.72 ± 0.03 (4)	1.06 ± 0.04 (7)^*^	----	1.67 ± 0.12 (11)
H (*n* = 37)	0.79 ± 0.00 (1)	1.97 ± 0.10 (36)^***^	----	2.78 ± 0.10 (37)

*: significant differences at *p* < 0.05

***: *p* < 0.000, as compared to LFTS

**Table 3:** Sensitivity and specificity of CCA and LFTS for each subgroup

	CCA			LFTS		
	L	M	H	L	M	H
Sensitivity	0.80	0.78	0.95	0.91	0.92	0.97
Specificity	0.82	0.82	0.95	0.90	1.00	1.00

### Distribution of Intensities Using LFTS and POC-CCA Strip

**Fig. 4:**
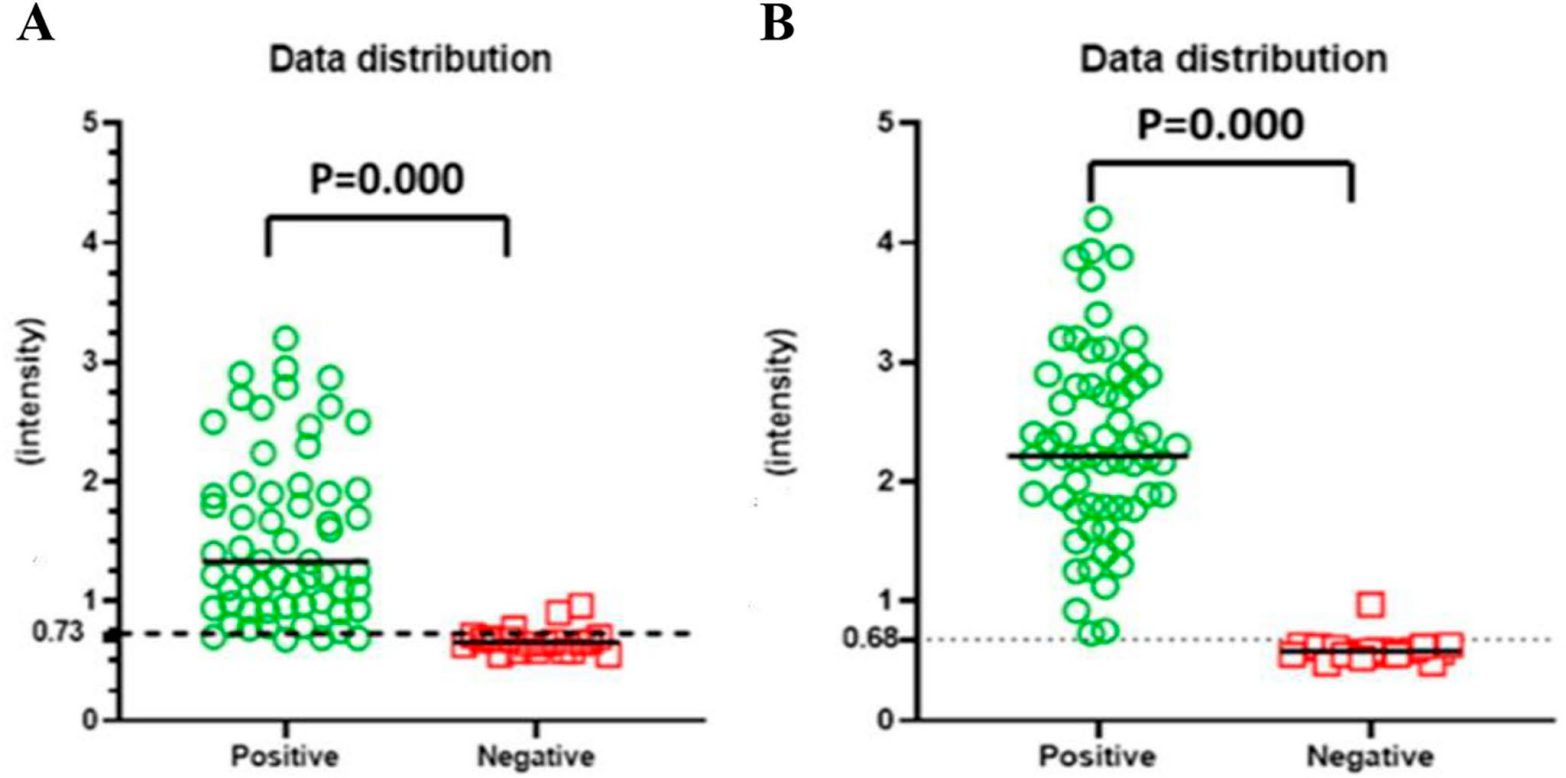
Distribution of color intensities of CCA strip (**A**) and LFTS (**B**) in urine samples. *P* < 0.000: Significant difference was found according to the Mann-Whitney test

**Fig. 5:**
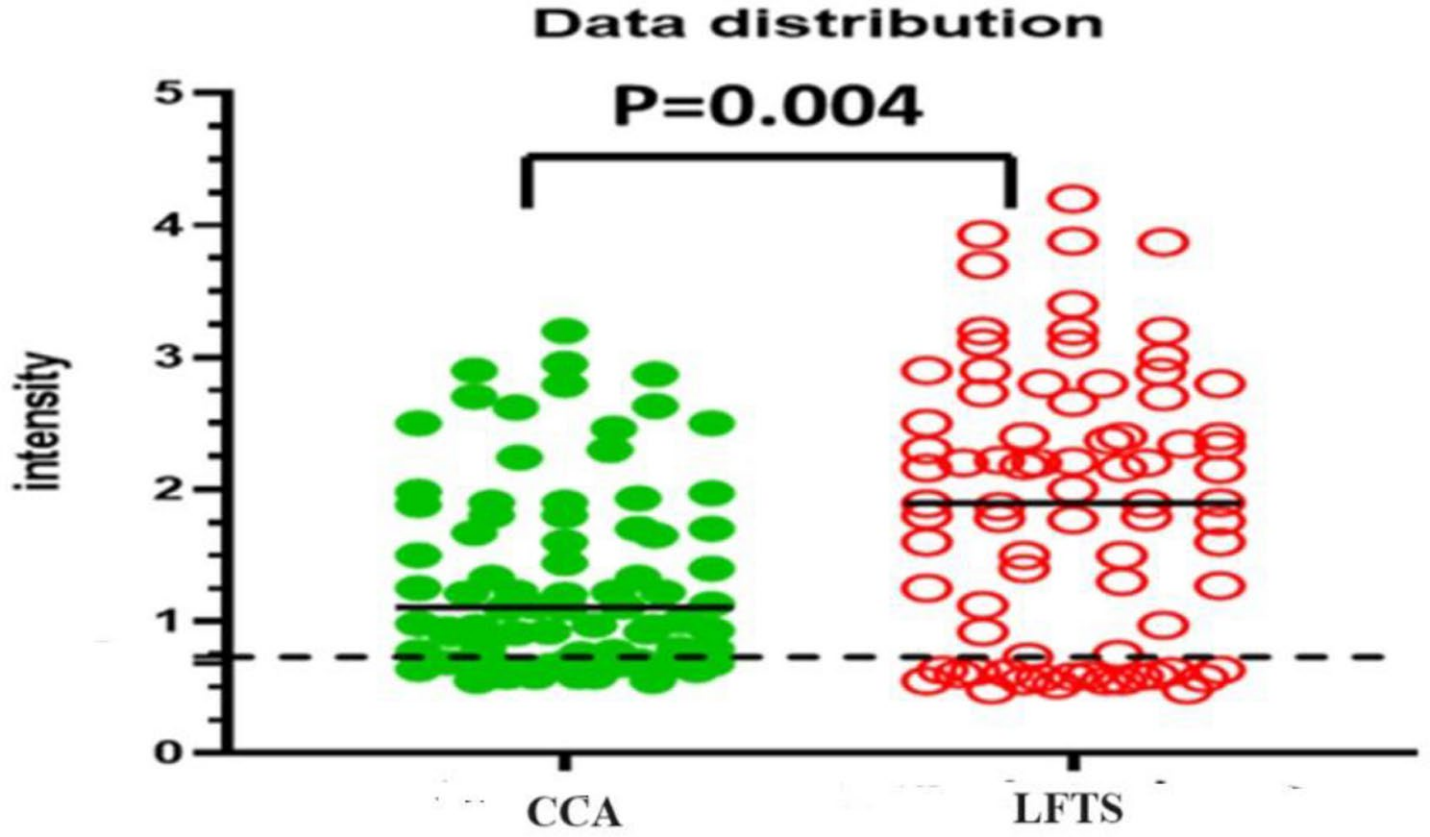
Distribution of color intensities of CCA strip and LFTS in urine samples. Compared to the CCA strip, the median value of intensity of the LFTS was significantly elevated (*p* = 0.004)

**Fig. 6:**
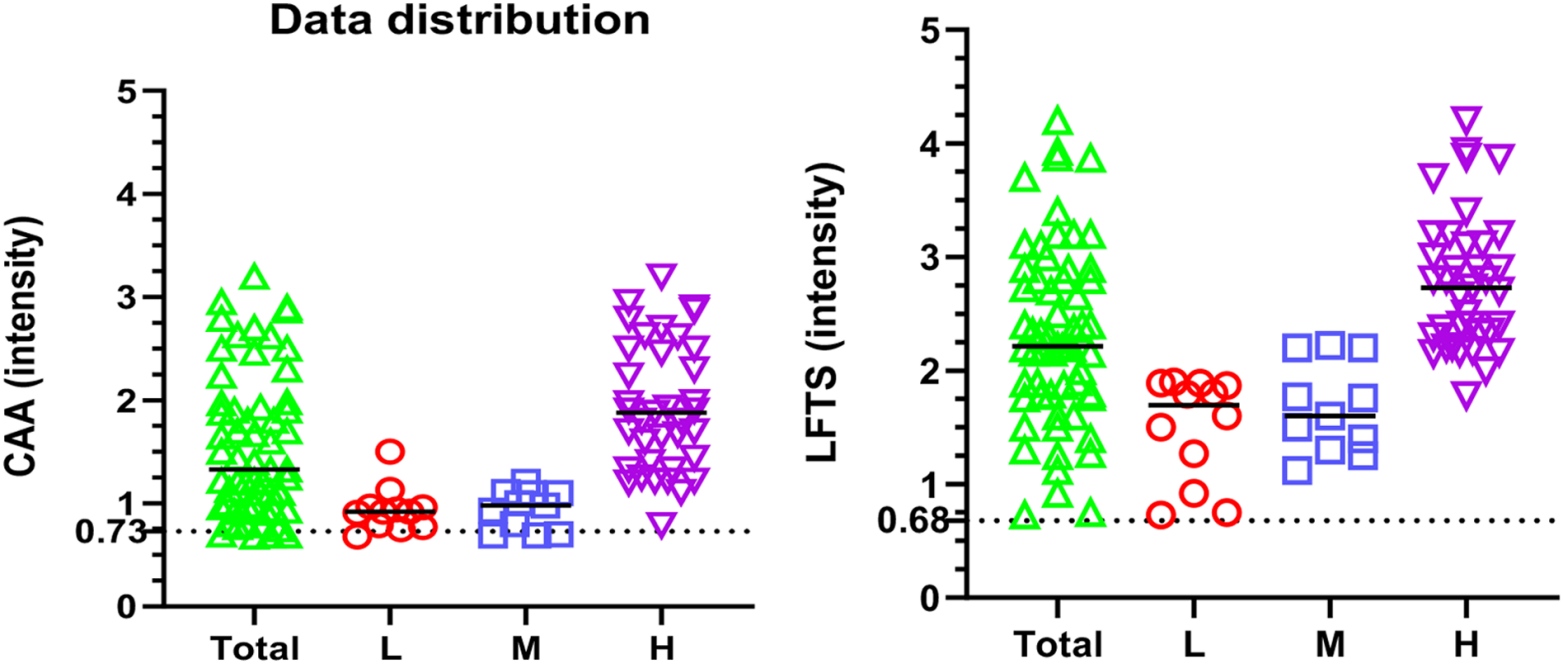
Distribution of color intensities of CCA strip and LFTS in different levels of infection. The intensity values by the LFTS were always significantly higher than those of CCA

**Fig. 7:**
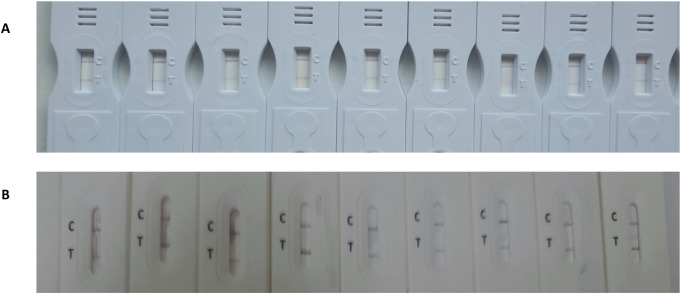
Comparison between CCA (**A**) and LFTS (**B**), some positive samples were tested by applying the same samples on both strips

The distribution of color intensities using LFTS and CCA strips is shown in Figs. 4 and 5, and 6. Both strips were able to differentiate between positive and negative results without any overlapping areas (*p* < 0.000) (Fig. 4). The median value of the LFTS positive test line intensity was significantly higher than that of CCA (*p* < 0.004), indicating higher resolution and better visual interpretation of the LFTS assay (Fig. 5). In cases of low, moderate, and heavy infections, the intensity values measured by the LFTS were consistently higher than those of CCA (Fig. 6). When positive samples were tested on both strips, the color intensity on the test band of our developed LFTS was found to be stronger than that on CCA using the same samples (Fig. 7).

## Discussion

Despite significant efforts, schistosomiasis remains a considerable health issue in certain countries, particularly in Africa [22, 23]. Many authors have mentioned that the sensitivity of the KK method is low, leading to false negative results due to various factors such as low parasitic burden, recent or chronic infections, post-treatment situations, or day-to-day variability in egg excretion in the host’s stool [24–26]. Therefore, immunological methods have recently emerged as a promising alternative, as they rely on direct antigen detection even before egg detection in the stool. The development of point-of-care (POC) tests for accurate screening is essential [8].

In our previous research, we successfully developed lateral flow tests (LFTS) for the rapid detection of *S. mansoni* antigen in urine samples. In this study, our objective was to assess and compare the effectiveness of our developed LFTS with the point-of-care circulating cathodic antigen (POC-CCA) kit. Samples (urine and stool) were collected from hot spot areas in Egypt, with the age range of the patients included in this study being between 12 and 30 years old, encompassing school-age children and young adults. This age group is of particular concern due to the concentration of the disease in children and economically active individuals [27, 28].

When both assays were tested on the same positive and negative cases, our developed LFTS exhibited a sensitivity and specificity of 96.7% and 95%, respectively, compared to the CCA strips that showed 85% and 90%, respectively. These results align with various other research works that have discussed the lower sensitivity of the POC-CCA assay [29, 30]. Furthermore, in 2021, Bezerra et al. reported that the sensitivity and specificity of POC-CCA varied according to the prevalence and intensity of the infection, and that the diagnostic accuracy of the CCA assay was diminished, resulting in a large number of false negative results in low-intensity infections [24]. Van Dam et al. (2004) also reported the lower sensitivity of POC-CCA in cases of light schistosomiasis infection [10]. Additionally, different research works have mentioned the cross-reactivity of the CCA assay, which contributes to its false positive results [31, 32].

In the current study, while 20% (12 cases out of 60) had a light infection (< 50 EPG) as determined by the KK method, only 2 cases (16.6%) showed negative results by the developed LFTS, compared to 4 cases (33.3%) that showed negative results by POC-CCA, which is consistent with other studies reporting high rates of false-negative results (low sensitivity) ranging from 55.6 to 58.3% and specificity from 76.9 to 78.4% [31–33]. Van Dam et al. (2004) reported the failure of POC-CCA to identify light infections in their study [10]. These results highlight the priority of using our developed LFTS for early diagnosis of schistosomiasis, as previously demonstrated by Kamel et al. (2019) [5]. There were 3 positive results considered false positive relative to KK (out of 20), with two (0.4%) being positive by CCA and one (0.2%) by LFTS. Furthermore, the intensity of the test line band, which is directly proportional to the concentration of the antigen in the sample, was consistently higher in the LFTS than in the CCA strip. As shown in our results, the color intensities observed in positive cases were always higher in the LFTS, even with low infection, compared to those of POC-CCA. This difference was statistically significant, as indicated by the elevated median values of color intensity in the LFTS.

(*p* = 0.004).

Both assays (LFTS & CCA) showed agreement with the intensity of infection (EPG), as reflected by the kappa value. For LFTS, the kappa value was 0.902, indicating an almost perfect agreement between the two assays, while for the CCA assay, it was 0.672. These findings are in line with the work of Ferreira et al. and Bezerra et al., who reported poor agreement (0.146 and 0.37, respectively) between the POC-CCA assay and the Kato-Katz technique [33].

In conclusion, based on the current study, the use of our developed LFTS, with its higher sensitivity and specificity, is recommended in Egypt as a reliable, rapid, and easy-to-perform point-of-care test to improve the diagnosis of active schistosomiasis, especially in cases of light infection, and to assist in infection control. However, additional assessment of the kit using a larger number of cases is recommended before applying it for routine diagnosis and screening studies.

## Data Availability

No datasets were generated or analysed during the current study.

## Abbreviations

KK: Kato-Katz
LFTS: Lateral flow test strip
POC-CCA: Point-of-care Circulating Cathodic Antigen detection
EPG: Eggs per gram of stool sample
TBRI: Theodor Bilharz Research Institute
ELISA: Enzyme-linked immunosorbent assay
FP: False positive
AuNPs: Gold nanoparticles
MAbs: Monoclonal antibodies
MCM: Mobile crystalline material
MSN: Mesoporous silica nanoparticles
RT: Room tempreture
BSA: Bovine serum albumin
PBS: Phosphate buffer solution
ROC: Receiver operating characteristic
AUC: Area under the curve
L: Light
M: Moderate
H: Heavy
LFIAs: Lateral flow immune assays
CSA: Circulating Schistosoma mansoni antigen
HRP: Horse raddish peroxidase
OPD: O-phenylene diamine dihdrochloride 10 mg (OPD)
SEA: S. mansoni soluble egg antigen
Gel Doc XR+: Gel documentation system
NIH: National Institutes of Health
